# MAFB: a key regulator of myeloid commitment involved in hematological diseases

**DOI:** 10.1038/s41420-025-02551-4

**Published:** 2025-06-12

**Authors:** Antonio Benedetto Ventura, Tiziana Loconte, Antonio Negri, Luigi Viggiano, Giuseppe Fiermonte, Sabino Ciavarella, Attilio Guarini, Giacomo Volpe

**Affiliations:** 1 Hematology and Cell Therapy Unit, IRCCS Istituto Tumori “Giovanni Paolo II”, Bari, Italy; 2 Department of Biology, University of Bari “Aldo Moro”, Bari, Italy; 3 Department of Bioscience, Biotechnology and Environment, University of Bari “Aldo Moro”, Bari, Italy

**Keywords:** Mechanisms of disease, Acute myeloid leukaemia

## Abstract

The MAFB protein, a member of the MAF family of bZip transcription factors, plays a pivotal role in various biological processes, including cell differentiation, development, and homeostasis. Characterized by its selective expression in monocytes and macrophages, MAFB has been shown to play a crucial role during myeloid lineage differentiation, acting as a critical determinant in the transition from multipotent progenitors to fully differentiated monocytes. By modulating the expression of genes associated with immune activation and inflammation, MAFB plays a vital role in maintaining immune homeostasis and responding to pathogenic challenges. Dysregulation of MAFB expression or function has been implicated in several pathological conditions, including hematological malignancies and metabolic disorders. In particular, aberrant MAFB activity has been associated with the progression of diseases such as multiple myeloma and acute myeloid leukemia as well as other solid tumors, where it may contribute to the survival and proliferation of malignant cells, thereby promoting disease progression. MAFB and downstream targets of its transcriptional network are now being regarded as predictive biomarkers for certain types of tumors as well as being considered as potential therapeutic targets for cancer treatment. In this review, we summarize current knowledge on both physiological and pathological roles of MAFB and highlight the impact of its deregulation on hematological cancer initiation and progression.

## Facts

- MAFB belongs to the MAF family of bZIP transcription factors.
- MAFB is a crucial regulator of the commitment of multipotent progenitors towards monocyte differentiation.
- MAFB acts as a repressor of erythroid maturation.
- MAFB is significantly deregulated in diverse type of cancer and often correlated with inferior outcomes.

## Open questions

- What are the potential players of the MAFB transcriptional network that could mediate its involvement in cancer?
- Can MAFB be regarded and exploited as an anti-cancer target to improve patient treatment?
- Why does MAFB have such diverse effect on hematological cancers?

## Introduction

The MAF family of oncogenes was first identified in the genome of the avian transforming retrovirus AS42 [1, 2]. Since their discovery, Maf-related proteins have been recognized across a wide range of species, demonstrating a conserved DNA-binding site that allows these proteins to function effectively as transcription factors. Maf transcription factors are integral to various biological processes, playing essential roles in the development, differentiation, and functional establishment of numerous organs, tissues, and cell types. The transcription factor MAFB plays a fundamental role in maintaining cellular identity and function across various biological systems. It is particularly essential in hematopoiesis, where it acts as a master regulator of monocytic commitment and macrophage differentiation, ensuring the proper balance between progenitor proliferation and terminal differentiation. Beyond hematopoiesis, MAFB contributes to tissue homeostasis in diverse organs, including the kidney [3], pancreas [4–6], lens [7–9], epidermis [10–12], osteoclasts [13] and cartilage [14, 15], highlighting their diverse functional repertoire. Dysregulation of MAFB has been implicated in multiple hematological malignancies, where it exerts contrasting roles depending on the disease context. In multiple myeloma, MAFB functions as an oncogene, driving tumor progression and therapy resistance. Conversely, in acute myeloid leukemia (AML), its expression is often suppressed, and its reactivation promotes myeloid differentiation, indicating a tumor-suppressive role. In this review, we will primarily focus on the MAFB and will discuss the molecular mechanisms by which it influences cellular differentiation and function. By synthesizing current research findings, this article aims to enhance our understanding of the multifaceted roles of MAFB proteins in homeostasis and disease, thereby laying the groundwork for potential therapeutic interventions targeting this critical regulator of gene expression.

## The MAF family and the discovery of MAFB

The Maf transcription factors belong to the AP1 superfamily of basic leucine zipper (bZip) transcription factors [16], a group that includes several oncogenes. The foundational discovery of the Maf family traces back to 1992 when the AS42 retrovirus was identified to harbor v-maf, a viral oncogene capable of inducing musculoaponeurotic fibrosarcoma in chickens [1]. In the same study, v-Maf was demonstrated to have the ability to transform chicken embryonic fibroblasts in culture, establishing the oncogenic potential of Maf proteins. Subsequent research by Fujiwara et al. employed cDNA library screens and led to the identification of two additional Maf family members, later named MafF and MafK [17]. Since then, the Maf family has expanded to include seven members, categorized into two subgroups based on molecular size: small Maf and large Maf transcription factors [18].

Small Maf proteins, including MafF, MafG, and MafK, typically range from 150 to 160 amino acids and lack a transactivation domain [17, 19]. This structural limitation renders them incapable of directly activating transcription, thus exerting regulatory influences by forming homodimers and repressing gene expression. Conversely, small Mafs can activate transcription by heterodimerizing with other Maf family members or other bZIP transcription factors that contain a transactivation domain. Through these interactions, small Maf proteins facilitate transcriptional activation or repression within a complex regulatory network [20]. As dimeric partners of the bZIP NF-E2/Nrf family, small Maf proteins play significant roles in antioxidant responses, potentially linking them to oncogenic pathways, although direct involvement in human cancer remains to be conclusively demonstrated.

In contrast, large Maf transcription factors, including MafA (or L-Maf), MafB, c-Maf, and Nrl, range from 240 to 340 amino acids and are defined by their bZip domains, which are essential for protein dimerization and DNA binding [9, 21–23]. In contrast to small Maf, these proteins contain an acidic TAD in their amino-terminal part. This structural motif allows large Maf proteins to function as potent transcriptional regulators, playing pivotal roles in cell differentiation, tissue development, and organogenesis. For example, the mouse Mafb gene is indispensable for hindbrain segmentation during embryogenesis, while c-Maf is broadly expressed across multiple tissues, including the liver, renal tubules, adipocytes, and muscle, indicating its diverse physiological roles. Unlike small Maf proteins, large Maf proteins have been directly implicated in carcinogenesis, with evidence spanning cell culture, animal models, and human cancers. A key distinguishing feature of large Maf proteins is their amino-terminal transactivation domain, which is essential for gene regulation [2, 23–26]. Maf proteins, akin to other AP1 superfamily members, recognize and bind DNA via their bZip domains at specific sequences known as Maf-recognition elements (MAREs) [18, 27–30]. These elements feature a palindromic TGCTGAC(G)TCAGCA sequence, further classified into two subtypes: t-MAREs, which incorporate a 12-O-tetradecanoyl phorbol 13-acetate (TPA)-responsive element (TRE) core (TGCTGACTCAGCA), and c-MAREs, containing a cAMP-responsive element (TGCTGACGTCAGCA)[18, 31]. The classical MARE structure is bound by the basic domain of Maf proteins, while an extended homology region is responsible for recognizing a TGC flanking sequence [27, 32–34]. Additionally, studies analyzing the α-crystallin gene bound by L-Maf, particularly those conducted by Yoshida and colleagues, revealed that Maf proteins can also bind DNA sequences containing only half of the palindromic MARE, provided these sequences are flanked by 5’-AT-rich regions [35, 36].

The identification of MafB in 1994 further expanded the understanding of the Maf oncogene family [23]. MafB encodes a 311-amino-acid protein characterized by a canonical bZip motif, sharing significant homology with v-Maf and other Maf-related proteins. Functionally, MafB exhibits the ability to homodimerize and bind MARE sequences with high specificity. It also forms heterodimers with v-Maf and Fos proteins, although it does not interact with Jun. Transient co-transfection assays have demonstrated that both v-Maf and MafB function as transcriptional activators of MARE-linked promoters, albeit with MafB exhibiting a lower transactivation potential than v-Maf. Notably, overexpression of mafB induces transformation in chicken embryo fibroblasts in vitro, reinforcing the oncogenic potential of Maf proteins [1, 23, 37].

The dual role of Maf proteins in normal physiology and oncogenesis underscores their significance in transcriptional regulation. Their involvement in key developmental pathways highlights their necessity in cell differentiation, while their oncogenic potential points to their ability to disrupt cellular homeostasis when dysregulated. The structural dichotomy between small and large Maf proteins, particularly regarding their transactivation domains, reflects their functional divergence, with small Maf proteins primarily serving as modulatory elements and large Maf proteins acting as direct transcriptional regulators. As part of the AP1 superfamily [38, 39], Maf proteins integrate complex regulatory signals, demonstrating the intricate balance required for their physiological functions and their contribution to oncogenic processes when this balance is disrupted. Continued research into the mechanistic underpinnings of Maf protein activity will provide further insights into their roles in development, differentiation, and cancer progression, with potential implications for targeted therapeutic strategies.

## The role of MAFB in hematopoiesis

**Fig. 1:**
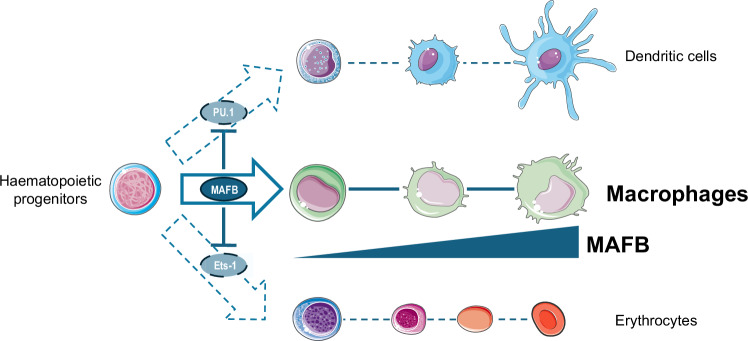
MAFB function and its interplay with other hematopoietic transcription factors. during myeloid maturation. Schematic representation of MAFB expression activation during myeloid commitment with concomitant downregulation of PU.1 and Ets-1 to prevent dendritic and erythroid fate decisions.

The transcription factor MafB has emerged as a critical regulator of hematopoiesis, particularly in myeloid differentiation and lineage commitment almost three decades ago [40, 41]. Pioneering studies by Sieweke et al. in the mid-1990s provided foundational insights into MafB’s role in hematopoietic differentiation, demonstrating its function as interaction partner of Ets-1 [40]. MafB’s direct binding to the DNA-binding domain of Ets-1 was shown to repress its activity, preventing the transcriptional activation of erythroid-specific genes and thereby inhibiting erythroid differentiation. This interaction was first identified using a yeast two-hybrid screen and later confirmed in avian cell models, leading to the demonstration that MafB bound the DNA-binding domain of Ets-1 and inhibited Ets-1-mediated transactivation of synthetic promoters containing Ets binding sites. Further experiments showed that ectopic expression MafB in erythroblasts inhibits transferrin receptor transactivation, a crucial regulator of erythroid differentiation, leading to reduced transferrin receptor expression and a consequent blockade in erythrocyte maturation [42]. Notably, these effects were not associated with altered cellular proliferation, indicating that MafB primarily modulates lineage fate rather than influencing growth kinetics (Fig. 1).

Subsequent studies established that MafB plays an essential role in myeloid lineage specification. Ectopic expression of MafB in primary avian hematopoietic progenitor cells transformed by the E26 virus demonstrated the requirement for MafB upregulation to permit the correct transition from hematopoietic stem cells to monocytes. This transition was characterized by an increased formation of myeloid colonies at the expense of erythroid commitment and resulted in the generation of functional macrophages with phagocytic activity capable of responding to lipopolysaccharide (LPS) stimulation. A dominant-negative mutant of MafB, lacking the N-terminal transactivation domain, disrupted this process by preventing endogenous MafB from binding its target genes. This dominant-negative MafB mutant severely impaired myeloid colony formation and blocked macrophage differentiation, providing direct evidence of MafB’s role in myeloid commitment [43].

Another milestone on the role of MafB in hematopoiesis and myeloid differentiation was set through studies on its interplay with PU.1, another key hematopoietic master regulator [44]. Using myeloblast cell models, Bakri et al. demonstrated the forced PU.1 expression skews differentiation toward dendritic cells, while MafB overexpression exclusively drives macrophage fate, thus underscoring the importance of maintaining a balanced PU.1-MafB axis to ensure appropriate myeloid lineage specification and immune function [45] (Fig. 1). Similarly, Saiga et al. highlighted the function of MAFB as negative regulator of the IRF-7/Spi-B-mediated transactivation of type I IFN genes in murine plasmacytoid dendritic cells [46].

Microarray analysis of human hematopoietic cells provided compelling evidence for the role of MAFB in guiding the commitment of myeloid progenitors toward monocytic differentiation. In fact, a striking upregulation of *MAFB* expression was observed in CD14+ monocytes compared to CD34+ hematopoietic stem/progenitor cells and other hematopoietic populations, highlighting its lineage-specific function [47]. To functionally validate this finding, retroviral overexpression of full-length *MAFB* cDNA was performed in human myeloid cell lines U937 and THP1, as well as in purified CD34+ cells derived from cord blood [48]. In both experimental settings, *MAFB* overexpression led to a significant increase in the number of phenotypic monocytes, reinforcing its essential role in promoting monocytic differentiation. While data on the essentiality of *MAFB* expression for monocytic commitment accumulated, it was still not clear what molecular mechanisms were governing its interaction with other myeloid transcription factors, such as MYB [49, 50], which promotes myeloid precursor proliferation cells at the expenses of their differentiation. In a pivotal study by Tillmanns and colleagues, it was demonstrated that post-translational SUMOylation of MafB at lysines 32 and 297 plays a critical role in balancing progenitor proliferation and macrophage differentiation. Specifically, SUMOylated MafB exhibits reduced transcriptional activity, thereby limiting its ability to counteract MYB-mediated differentiation blocks. In contrast, non-SUMOylated MafB can effectively override MYB’s inhibitory influence, facilitating macrophage maturation through cell cycle arrest and the activation of lineage-specific gene programs [51].

Beyond its role in lineage commitment, further studies revealed that MafB can also regulate the self-renewal potential of myeloid cells in cooperation with other Maf partners. In fact, Aziz et al. demonstrated that combined deletion of MafB and c-Maf in myeloid cells resulted in the acquisition of self-renewal properties, allowing monocyte-derived cells to form discrete colonies in semi-solid culture conditions when supplemented with macrophage colony-stimulating factor (M-CSF). These self-renewing monocytes could be serially transplanted and expanded in vitro without the acquisition of tumorigenic characteristics, suggesting that MafB functions as a gatekeeper preventing aberrant self-renewal in differentiated myeloid cells [52, 53]. Mechanistically, the loss of MafB and c-Maf enables monocyte-to-macrophage precursors to re-enter the cell cycle via the activation of c-Myc and KLF4, two transcription factors associated with stemness and cellular plasticity.

Intriguingly, MafB deletion also influences hematopoietic stem cell (HSC) dynamics. MafB-deficient long-term HSCs (LT-HSCs) exhibit enhanced proliferation and a competitive advantage in bone marrow reconstitution assays. In serial transplantation experiments, MafB-deficient LT-HSCs efficiently repopulated the myeloid compartment while maintaining their ability to contribute to T cell and other hematopoietic lineages. Notably, despite their increased proliferative capacity, these cells did not display signs of exhaustion or uncontrolled self-renewal. Further mechanistic studies revealed that MafB deficiency sensitized HSCs to M-CSF signaling by upregulating PU.1 expression, thereby reinforcing myeloid lineage commitment at the expense of alternative fates [54].

Collectively, these findings underscore the multifaceted role of MafB in hematopoiesis, influencing myeloid differentiation, proliferation, and lineage fate decisions. The intricate interplay between MafB, Ets-1, PU.1, and Myb is essential for balancing erythroid and myeloid differentiation, while post-translational SUMOylation fine-tunes its transcriptional activity. The ability of MafB-deficient cells to re-enter a self-renewing state suggests a key role in regulating differentiation pathways that may have implications in regenerative medicine and hematological disorders. Future research should explore the therapeutic potential of modulating MafB activity in hematopoietic stem cells and myeloid progenitors to enhance immune cell function and address hematopoietic malignancies.

## MAFB in hematological diseases

Since the original identification of v-Maf transforming ability in chicken embryonic fibroblasts in culture and its role in musculoaponeurotic fibrosarcomagenesis in vivo, a substantial body of evidence has established cellular Mafs, including MafA and MafB, as bona fide oncogenes. To further understand their paradoxical roles in both terminal maturation and tumorigenesis, Pouponnot et al. conducted a comparative study assessing both the transforming and the transactivating capacities of MafA and MafB [55]. Their findings revealed that while MafA induced cell transformation to both growth-factor-independent and anchorage-independent conditions, MafB was characterized by less potent transforming activity. This difference was attributed to MafB’s lower expression levels and greater instability compared to other Maf transcription factors, suggesting that Maf-driven oncogenesis would require high oncogene expression, as it was the case for transgenic mouse models of T cell lymphoma with high MAF transgene copy numbers [56, 57]. Importantly it was observed that such oncogenic potency was cell context specific and proposed that those protein could function both as oncogenes and tumor suppressors [55, 58–62].

A major contribution to understanding Maf protein-mediated oncogenesis has come from studies on multiple myeloma (MM), a malignancy arising from antibody-secreting mature B cells [63, 64]. Several primary and complex secondary translocations in MM have been reported to involve MAF family members [65–67]. Among the most recurrent ones, the t(14;16)(q32.3;q23) translocation, first described in the late 1990s, was shown to involve c-Maf and the IgH (14q32) or IgL loci, confirmed in a panel of MM cell lines and primary samples [68]. Soon after, Hanamura et al. performed a comprehensive fluorescence in situ hybridization analysis of the 20q11 breakpoints involved in the t(14;20) translocation in 16 different MM cell line, observing that this alteration resulted in the ectopic expression of *MAFB*, this being potentially mediated by the 3′α enhancers of the IgH gene [69]. This translocation, confirmed and characterized through molecular cloning, was found in approximately 2% of MM cases, while overall MAF translocations account for 8–10% of cases, with MAF in 5% and MAFA in about 1% of cases [70–74].

Although MAFB is translocated in only 5% of MM cases, its overexpression is detected in nearly 50% of MM patients, correlating with poor clinical outcomes [75–79]. To date, the mechanisms underlying its overexpression in the absence of genetic lesions are still to be fully elucidated. In the pursuit of this, work from Vicente-Duenas based on the generation of MafB transgenic mice demonstrated that targeting the expression of *MafB* to mouse B cells did not result in any disease onset. Conversely, targeting *MafB* transgenic expression to hematopoietic stem progenitor cells (HSPCs) would reprogram the cells through epigenetic mechanisms and lead to development of plasma cell neoplasias in vivo, demonstrating that transgenic HSPCs displayed a molecular profile more closely resembling that of B cells and tumor plasma cells than any other cells, including normal HSPCs [80]. To assess whether MAFB mutations associated to specific disease categories or patient subgroups, a whole exome sequencing study was conducted on 463 patients from the Myeloma XI cohort in the UK, revealing that mutations occurring in the gene bodies of MAF and MAFB were significantly linked to those patients exhibiting an APOBEC mutational signatures [81], suggesting that MAFB mutations are associated or may contribute to a more aggressive MM behavior [82].

**Fig. 2:**
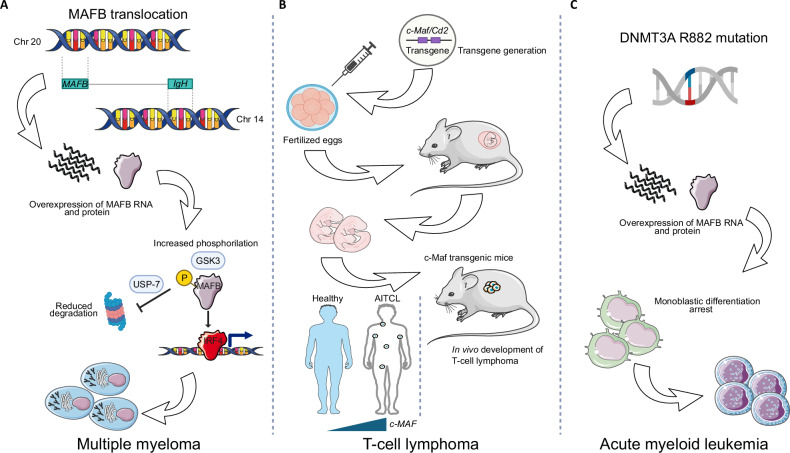
MAF proteins involvement in hematological diseases. **A** MAFB overexpression in multiple myeloma characterized by the t(14;20)(q32;q11) translocation. **B** Generation of a transgenic mouse to study the role of c-Maf in T cell lymphoma genesis and comparison with *c-MAF* expression levels in patients with angioimmunoblastic T cell lymphoma (AITCL). **C** Overexpression of MAFB in acute monoblastic leukemias characterized by DNMT3A R882 mutation.

Herath et al. demonstrated that both MAFB and c-MAF are phosphorylated by Ser/Thr kinase GSK3 on residues T62 and T58, which enhances their transactivation capacity and showed that GSK3 inhibition via lithium chloride leads to their degradation in human MM cell lines [83, 84]. Additionally, it was shown that treatment with proteasome inhibitor bortezomib stabilizes MAF proteins with its consequent accumulation rather than degradation, explaining at least in part why bortezomib treatment displays low efficacy in MM patients bearing MAF translocations [83]. Further research by Abe and coworkers, by culturing MM cells in either normoxic and hypoxic conditions, demonstrated a strong correlation between MAFB and heme oxygenase-1 (HMOX1), a gene highly expressed in MM cells. Knockdown of *HMOX1* attenuated hypoxia-induced proteasome inhibitor resistance, leading to higher ROS levels and enhanced bortezomib effect [85]. On a similar note, mass spectrometry analysis of ubiquitination-associated proteins that are part of MAFB interactomes, led to the identification of the ubiquitin-specific protease USP7,a gene largely upregulated in myeloma cells and negatively associated with myeloma patients’ survival, as a principal interaction partner not only of MafB but also MafA and c-Maf [86]. USP7 was found to enhance their transcriptional activity and prevent them from being degraded and, consistently, silencing of USP7 resulted in Maf protein degradation with increased polyubiquitination levels and induction of apoptosis in MM cells [86] (Fig. 2A). Similar work conducted on the deubiquitinase USP5 demonstrated that the selective inhibition of this gene resulted in the induction of myeloma cell apoptosis mediated by the degradation of c-Maf but not of other MAF proteins [87].

A recent study by Katsarou and colleagues demonstrated that MAF acts as a pioneer transcription factor in multiple myeloma by mediating a global reshaping of chromatin accessibility and establishing a myeloma-specific transcriptional program that promotes tumor progression. In fact, it was shown that MAF proteins sustain myelomagenesis by cooperating with plasma cell specific transcription factor IRF4 and regulating the activation of super-enhancers that were previously inactive in healthy plasma cells [88]. A study by Van Stralen and colleagues aimed at identifying downstream transcriptional targets of upregulated MAFB in MM employed cDNA microarray profiling in U266 and UM1 MM cell lines, highlighting a set of 14 genes that appeared to be commonly regulated by both MAFB and c-Maf. These included *ANG*, *BLVRA*, *NOTCH2* and its downstream effectors *HES1*, *HES3* and *HES5*, as well as *ITGB7*, *ARID5A*, *CCR1* and *CCND2*, several of which have demonstrated functional relevance in MM pathogenesis and clinical outcome [89]. Among these, *CCND2*, that is typically upregulated by MAFB and overexpressed in MM, enhances cell proliferation by promoting G1-S cell cycle transition. This was validated by pharmacological targeting studies in which kinetin riboside mediated silencing of *CCND2* induced cell cycle arrest and triggered tumor cell-selective apoptosis as well as suppressing myeloma growth in xenograft models [90]. In parallel, Neri et al. reported that the silencing of *ITGB7* impaired MM cell adhesion to extracellular matrix and reversed drug resistance enhancing the response to bortezomib and melphalan [91]. Additionally, the inhibition of *CCR1* by small molecule inhibitors MLN3897 or BX471 reduced migration and homing of MM cells to the bone, thus limiting skeletal damage, and improved the therapeutic efficacy of bortezomib [92, 93].

While a large flurry of publication has assessed the function of MAFB in MM, its involvement in lymphoma and leukemia is largely understudied. The only hint of involvement of MAF proteins in lymphoma comes from a study in which Morito and coworkers generated a transgenic mouse to overexpress *c-Maf* under the control of the CD2 promoter. In these experimental settings, it was demonstrated that overexpression of *c-Maf* skewed T cell differentiation leading those mice to develop angioimmunoblastic T cell lymphoma in vivo, thus identifying c-Maf as an oncogene for T cell malignancies [56, 57]. To date however, no available studies provide any evidence that suggest an oncogenic role for MAFB in lymphomagenesis (Fig. 2B).

**Fig. 3:**
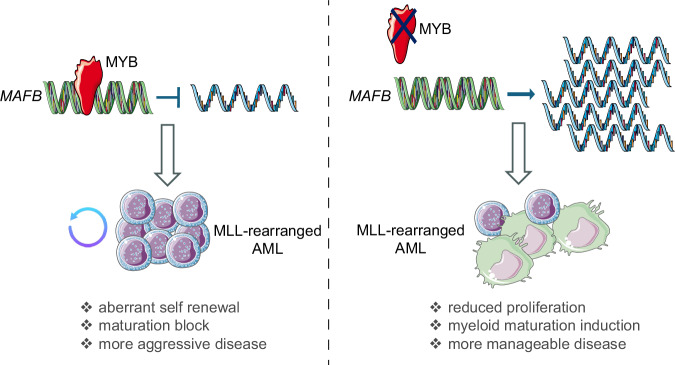
Interplay between MYB and MAFB in MLL-rearranged AML. Schematic representation showing MYB direct recruitment to MAFB promoter to repress it, resulting in more proliferative and aggressive disease (left panel). Ablation of MYB expression leads to reactivation of MAFB expression with a consequent block of proliferation and activation of myeloid maturation.

The association of MAFB with acute myeloid leukemia (AML) was originally suggested when it was found to be overexpressed in a set of patients with acute monoblastic leukemia [94]. Furthermore, Yang et al. found that the DNMT3A R882 mutation in AML is associated with increased expression of *MAFB*, which contributes to a monocytoid (M4/M5) immunophenotype in AML blasts [95] (Fig. 2C). Importantly, MAFB was reported to be specifically targeted in NPM1 mutant leukemia through the overexpression of the long noncoding RNA LONA which, however, displayed different effects on myeloid maturation depending both on its nuclear or cytoplasmic localization and on the bases of NPM1 mutational status [96]. The idea that the mutational status could impact on the way MAFB influences monocytic commitment or reverse the maturation block and the disease outcome was further reinforced in a study conducted in our laboratory, in which we assessed the specific interplay between MAFB and another master regulator of hematopoiesis, namely MYB [49, 50, 97–99]. In this study, silencing of *MYB* led to very different phenotypic responses in different mutant settings, with the strongest phenotype being observed in MLL-rearranged leukemias, this being accompanied by a substantial derepression of *MAFB*. Importantly, we demonstrated that ectopic expression of *MAFB* could essentially phenocopy the effect of *MYB* ablation, leading to remarkable boost in the expression of mature myeloid cell markers, although this effect was restricted to MLL-rearranged leukemic cells only, while no effect was observed in the other leukemia classes tested, that is those characterized by either complex karyotypes or t(8;21) translocations [100]. In this study we hypothesized that MYB would exert its inhibitory function by directly repressing MAFB promoter (Fig. 3).

It is important to consider that the impact of MAFB expression varies depending on the hematological malignancy, largely due to the cell type-specific roles of MAFB, and the interplay with other transcription factors in balancing differentiation and proliferation. In multiple myeloma, MAFB acts as an oncogene, promoting tumor progression by enhancing cell survival, immune evasion, and resistance to therapy. In contrast, in AML *MAFB* overexpression enforces monocytic differentiation, pushing leukemic blasts toward a more mature and less proliferative state, thereby reducing the aggressive nature of the disease. This dual role highlights the context-dependent function of MAFB, where its ability to drive differentiation in myeloid cells becomes a tumor-suppressive mechanism in leukemia, whereas its role in plasma cell malignancies enhances oncogenesis.

## Strategies of pharmacological targeting of MAFB

It has become evident that MAFB can be regarded as a therapeutic target amenable to manipulation although this represents a complex challenge as its modulation must be tailored on the bases of what disease we intend to treat. Given that the high expression of *MAFB* is usually observed to contribute to disease establishment and progression in myeloma, therapeutic approaches should focus on its downregulation. Conversely, the treatment of specific subtypes of AML would require *MAFB* upregulation to block leukemic proliferation and induce myeloid maturation. Despite its potential as a target, no selective inhibitors or antagonists specifically designed to inhibit MAFB have been reported to date. A recent compound screening in the pursuit of small molecule inhibitors that could target c-Maf transcriptional activity has suggested that some tested molecules were also efficient in repressing MAFB transcriptional activity, although no further data have been reported in this matter [101]. Our recent work has suggested that a boost in *MAFB* expression is required to reverse the leukemic phenotype and restore myeloid commitment in specific subtypes of leukemias, thus requiring the identification of new agonistic molecules that could be fit for this purpose. Two independent approaches targeting MYB activity in AML have shown promising data in increasing MAFB levels in MLL-rearranged AML, those being Sodium Monensin [102] and a peptidomimetic inhibitor termed MYBMIM [103]. Future studies are required to test those compounds to determine their suitability for AML treatment.

Recent studies into macrophage reprogramming in inflammatory settings have also revealed a potential link between MAFB and inflammatory response modulation. In fact, the LXR inhibitor GSK2033 has been shown to control macrophage polarization through upregulation of *MAFB* [104, 105]. We have tested this inhibitor in our laboratory with the idea of searching for new routes to phenocopy *MAFB* ectopic expression in MLL-r leukemia, however we could only obtain a modest raise in *MAFB* levels which were insufficient to significantly impact on the disease phenotype (personal communication).

Another promising avenue has emerged from research into macrophage reprogramming in rheumatoid arthritis. The JAK inhibitor Upadacitinib was found to induce a robust increase in *MAFB* expression, promoting a macrophage phenotype associated with inflammation resolution [106]. This finding suggests that JAK inhibition could be explored as a novel strategy for MAFB induction in AML or other conditions where its expression is therapeutically beneficial. The list of compounds that have been reported to have a direct or indirect effect on MAFB is provided in Table 1. Further research is warranted to refine these approaches and develop effective compounds that can either suppress or enhance MAFB activity depending on disease context, thereby paving the way for more targeted and personalized therapeutic interventions.

**Table 1:** List of compounds and small molecule inhibitors with reported direct or indirect effects on MAFB expression or transcriptional activity.

Compound	Inhibited target	Effect on MAFB	Outcome	Reference
**IBS003214**	c-Maf	Reduction of transcriptional activity	Inhibition of proliferation in multiple myeloma cell lines	Asano et al. [101]
**IBS007125**	c-Maf	Reduction of transcriptional activity	Inhibition of proliferation in multiple myeloma cell lines	Asano et al. [101]
**Monensin**	MYB	Gene expression activation	Inhibition of proliferation and induction of myeloid commitment in MLL-rearranged leukemia cell lines	Yusenko et al. [102]
**MYBMIM**	MYB	Gene expression activation	Inhibition of proliferation and induction of myeloid commitment in MLL-rearranged leukemia cell lines	Takao et al. [103]
**GSK2033**	LXRα	Overexpression	Macrophages reprogramming in inflammatory conditions	De la Aleja et al. [104]
**Upadacitinib**	JAK2	Overexpression	Macrophages reprogramming in inflammatory conditions	Lopez-Navarro et al. [106]

## Conclusions

Over three decades of research on MAFB since its original discovery have accumulated substantial evidence about its pivotal role in homeostatic hematopoiesis, guiding monocytic commitments and macrophage differentiation while restraining excessive progenitor proliferation and suppressing both dendritic cells maturation and erythropoiesis. However, its role in hematological diseases can be highly context dependent, displaying contrasting functions, acting as an oncogene in some settings and as a tumor-suppressor in others. In fact, in MM and in T cell lymphoma MAFB overexpression is frequently associated with disease progression and dismal prognosis, promoting tumor cell survival, immune evasion and therapy resistance. Genomic translocations and epigenetic deregulation contribute to its aberrant expression, making MAFB a potential therapeutic target for disease modulation. Although very little work has been done on MAFB in the context of AML, recent work suggests that MAFB exhibits a tumor-suppressor role, although this evidence might be restricted to some specific leukemia subclasses, such as the leukemias with MLL-rearrangements. In these settings, forced expression of *MAFB* counteracted the leukemic enforcement driven by MYB, inducing cell cycle arrest and myeloid differentiation. Therapeutic strategies aiming at restoring *MAFB* expression in AML should be considered, perhaps using small molecule inhibitors like LXR or JAK inhibitors, those latter emerging as potential candidates to harness its tumor-suppressive function. Thus, while targeting *MAFB* downregulation is a rational approach for myeloma and lymphoma treatment, enhancing *MAFB* expression could serve as a differentiation therapy for AML. Future research should focus on developing selective MAFB modulators, tailored to its disease-specific role, to exploit its therapeutic potential while minimizing unintended effects on normal hematopoiesis.
